# Transient severe distributive shock due to early dumping syndrome: a case report

**DOI:** 10.1186/s13256-018-1800-2

**Published:** 2018-09-13

**Authors:** Jun Takeshita, Kei Nishiyama, Satoru Beppu, Nozomu Sasahashi, Nobuaki Shime

**Affiliations:** 1 Department of Intensive Care Medicine, Osaka Prefectural Hospital Organization, Osaka Women’s and Children’s Hospital, 840 Murodo-cho, Izumi, Osaka, 594-1101 Japan; 2 Department of Emergency and Critical Care Medicine, National Hospital Organization, Kyoto Medical Center, 1-1 Fukakusa, Mukaihata-cho, Fushimi-ku, Kyoto, 612-8555 Japan; 3 Department of Emergency and Critical Care Medicine, Institute of Biomedical & Health Sciences, Hiroshima University, 1-2-3 Kasumi, Minami-ku, Hiroshima, 734-8551 Japan

**Keywords:** Early dumping syndrome, Gastrectomy, Hypotension, Hyperlactatemia, Norepinephrine

## Abstract

**Background:**

Early dumping syndrome characterized by palpitation, dizziness, cold sweat, feebleness, and abdominal symptoms, occurs within 30 minutes after meals in patients who have undergone gastrectomy. This case report describes the case of a patient who presented with severe distributive shock due to early dumping syndrome; he recovered within a few hours after massive fluid infusion and vasopressor administration.

**Case presentation:**

Our patient was a 68-year-old Japanese man who underwent total gastrectomy for gastric cancer and was diagnosed as having late dumping syndrome. On admission, he developed severe shock and was treated with massive fluid administration. Based on the history of the present illness, past medical history, normal findings of blood chemistry test, transient course, and Sigtad score, which helps diagnose dumping syndrome, early dumping syndrome was considered the cause of severe distributive shock.

**Conclusions:**

Early dumping syndrome can cause severe shock requiring massive fluid infusion and vasopressor administration. It should be considered a cause of severe distributive shock in patients who have undergone gastrectomy.

## Background

Early dumping syndrome occurs within 30 minutes after meals in patients who have undergone gastrectomy. It is characterized by palpitation, dizziness, cold sweat, feebleness, and abdominal symptoms, all of which are due to hypotension [1–4]. However, there are no reports about severe hypotension requiring massive infusion and vasopressor administration. Here, we report the case of a patient who presented with severe distributive shock due to early dumping syndrome and recovered within a few hours after massive fluid infusion and vasopressor administration.

## Case presentation

A 68-year-old Japanese man, who had a history of total gastrectomy for gastric cancer and transcatheter arterial embolization for left adrenal gland aneurysm rupture, had been transported to our emergency department by ambulance several times. He had a history of repeated hypoglycemia after meals, leading to a diagnosis of late dumping syndrome. Prior to the most recent admission, he had abdominal pain followed by diarrhea after breakfast at approximately 8:30 a.m. He was found unconscious sitting on the toilet seat at approximately 9:00 p.m. and was transported to our emergency department 20 minutes later.

On arriving at our hospital, he was unable to describe his symptoms. His vital signs were as follows: Glasgow Coma Scale score, E3V4M6; respiratory rate, 30 breaths/minute; oxygen saturation, 99% under room air; blood pressure, 60/28 mmHg; heart rate, 90 beats/minute; and body temperature, 36.1 °C. Arterial blood gas analysis revealed metabolic acidosis with respiratory compensation, hyperglycemia, and hyperlactatemia (Table 1). Blood biochemistry findings were within the normal limits (Table 2). After rapid administration of 2000 mL of bicarbonate Ringer’s solution, his systolic blood pressure transiently increased to 100 mmHg, but this increase was not sustained. A chest radiograph and computed tomography images of his brain and whole body revealed no abnormal findings. Ultrasonography revealed normal contractility of his heart and collapse of the inferior vena cava. He was transferred to our intensive care unit (ICU) with further administration of bicarbonate Ringer’s solution.

**Table 1:** Arterial blood gas analysis (fraction of inspired oxygen: room air)

PaO_2_	110	Torr
PaCO_2_	15.8	Torr
HCO_3_^−^	8.4	mEq/L
BE	− 15.7	mEq/L
pH	7.344	
BS	289	mg/dL
Lac	10.6	mmol/L

*BE* base excess, *BS* blood sugar, *HCO*_*3*_^*−*^ bicarbonate ion, *Lac* lactate, *PaO*_*2*_ partial pressure of oxygen in arterial blood, *PaCO*_*2*_ partial pressure of carbon dioxide in arterial blood

**Table 2:** Blood biochemistry findings

TP	7.1	g/dL	Na	140	mEq/L
Alb	3.8	g/dL	K	3.0	mEq/L
CRP	0.02	mg/dL	Cl	103	mEq/L
PCT	0.053	ng/mL	WBC	4600	/μL
BUN	13	mg/dL	Hb	12	g/dL
CRE	1.17	mg/dL	Hct	37.9	%
Glu	286	mg/dL	PLT	19.4	10^4^/μL

*Alb* albumin, *BUN* blood urea nitrogen, *Cl* chloride, *CRP* C-reactive protein, *CRE* creatinine, *Glu* glucose, *Hb* hemoglobin, *Hct* hematocrit, *K* potassium, *Na* sodium, *PCT* procalcitonin, *PLT* platelet, *TP* total protein, *WBC* white blood cell

In the ICU, his lactate was decreased to 7.4 mmol/L, while the hypotension persisted. A central venous catheter was inserted into the right internal jugular vein, and continuous infusion of noradrenaline was started and increased to 0.13 μg/kg per minute. Antibiotics were not administered as neither blood chemistry nor imaging revealed any findings of infection. As his hemodynamics gradually stabilized, after 3500 mL of fluid administration, continuous infusion of noradrenaline was stopped 4 hours after the initial infusion. He was able to eat supper on the same day and was discharged from the ICU on the following day. During admission, the plasma cortisol level was found to be normal; therefore, no steroids were administered.

## Discussion and conclusions

To the best of our knowledge, this is the first report to describe severe distributive shock due to early dumping syndrome treated with massive infusion and vasopressor administration.

The pathogenesis of early dumping syndrome is as follows [1–7]. First, after gastrectomy, foods directly flush into the small intestine, causing sudden increases in osmotic pressure in the small intestine, followed by shifting of fluids from the extracellular spaces to the intestinal lumen. Second, excessive secretion of gastrointestinal hormones (bradykinin, serotonin) cause splanchnic vasodilatation. Together, these two components could cause distributive shock. Severe hypotension requires massive fluid loading with noradrenaline to stabilize hemodynamics. In this patient, there was no evidence suggesting other causes of distributive shock including septic origin, which further supported our hypothesis. The presence of abdominal symptoms (abdominal pain and diarrhea) also contributed to the diagnosis of early dumping syndrome, as well as the severe shock manifestation.

Patients who have late dumping syndrome are often revealed to have early dumping syndrome as well [8]. In our case, our patient’s history of total gastrectomy and late dumping syndrome, as well as his high Sigtad score (> 7) [3], which is used to diagnose dumping syndrome, also supported the diagnosis of early dumping syndrome.

Early dumping syndrome can cause severe shock that requires massive fluid infusion and vasopressor administration. It should be considered one of the causes of severe distributive shock in patients with a history of gastrectomy.

## Abbreviations

ICU: Intensive care unit
